# Continuous roadmapping in liver TACE procedures using 2D–3D catheter-based registration

**DOI:** 10.1007/s11548-015-1218-x

**Published:** 2015-05-20

**Authors:** Pierre Ambrosini, Daniel Ruijters, Wiro J. Niessen, Adriaan Moelker, Theo van Walsum

**Affiliations:** Biomedical Imaging Group Rotterdam, Erasmus MC, Rotterdam, The Netherlands; Department of Radiology and Medical Informatics, Erasmus MC, Rotterdam, The Netherlands; Interventional X-ray Innovation, Philips Healthcare, Best, The Netherlands; Imaging Science and Technology, Faculty of Applied Sciences, Delft University of Technology, Delft, The Netherlands; Department of Radiology, Erasmus MC, Rotterdam, The Netherlands

**Keywords:** Roadmap, 2D/3D Registration, Single-plane X-ray fluoroscopy, 3DRA, Abdominal, Breathing compensation

## Abstract

**Purpose:**

Fusion of pre/perioperative images and intra-operative images may add relevant information during image-guided procedures. In abdominal procedures, respiratory motion changes the position of organs, and thus accurate image guidance requires a continuous update of the spatial alignment of the (pre/perioperative) information with the organ position during the intervention.

**Methods:**

In this paper, we propose a method to register in real time perioperative 3D rotational angiography images (3DRA) to intra-operative single-plane 2D fluoroscopic images for improved guidance in TACE interventions. The method uses the shape of 3D vessels extracted from the 3DRA and the 2D catheter shape extracted from fluoroscopy. First, the appropriate 3D vessel is selected from the complete vascular tree using a shape similarity metric. Subsequently, the catheter is registered to this vessel, and the 3DRA is visualized based on the registration results. The method is evaluated on simulated data and clinical data.

**Results:**

The first selected vessel, ranked with the shape similarity metric, is used more than 39 % in the final registration and the second more than 21 %. The median of the closest corresponding points distance between 2D angiography vessels and projected 3D vessels is 4.7–5.4 mm when using the brute force optimizer and 5.2–6.6 mm when using the Powell optimizer.

**Conclusion:**

We present a catheter-based registration method to continuously fuse a 3DRA roadmap arterial tree onto 2D fluoroscopic images with an efficient shape similarity.

## Introduction

**Fig. 1:**
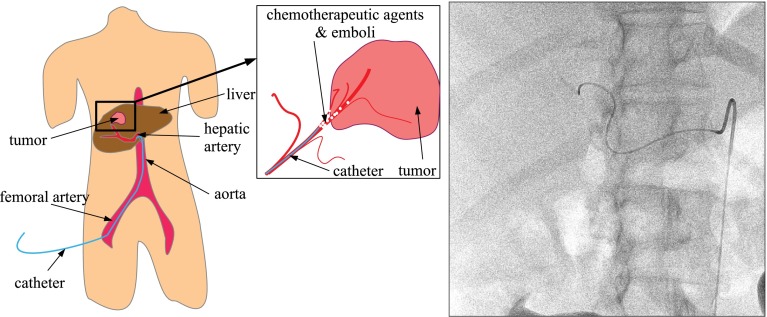
TACE intervention overview (*left*) and fluoroscopy example (*right*)

Transcatheter arterial chemoembolization (TACE) is a minimally invasive procedure to treat liver cancer (mostly hepatocellular carcinoma). In this procedure, a catheter is navigated toward a tumor via the femoral and hepatic artery, after which chemotherapeutic agents are injected. Currently, the interventionalist guides the catheter using single-plane 2D X-ray (fluoroscopy), visualizing only the catheter (Fig. 1). Frequently, contrast is injected to visualize the arteries. Computed tomography angiography (CTA) or 3D rotational angiography (3DRA) are used pre/perioperatively to visualize the tumor and feeding arteries. The navigation of the catheter using only 2D fluoroscopy is hampered by the inability to continuously visualize the arterial tree.

Purpose of our work is to integrate information of the vasculature from pre/perioperative 3D images by fusing it with the intra-operative 2D X-ray images. Such an approach enables a continuous up-to-date roadmap and thus may improve the guidance during the procedure and consequently has the potential to reduce intervention time, radiation dose and contrast agent use.

2D–3D registration for improving image guidance has been studied in cardiac, cranial, abdominal and orthopedic procedures. An overview of 2D–3D registration methods is presented by Markelj et al. [13] and Liao et al. [10]. Following [13], 2D–3D registration methods can be classified as extrinsic, intrinsic and calibration based. Extrinsic methods use markers to register and update the registration [17]. Usually objects visible on X-ray (e.g., small beads) are inserted close to the region of interest before 3D image acquisition. Intrinsic methods rely on anatomical structures such as bones or the vasculature and are generally intensity, gradient, feature based or a combination of them [13]. In abdominal interventions, the vasculature and catheters are mostly the only structures visible on 2D X-ray images that can be used for registration. In cardiac [3, 4, 14, 21, 22], cranial [7, 15, 26, 27] and abdominal [5, 8, 9] interventions, vessel-based registration have been used between pre- or perioperative 3D/4D CTA (computed tomography angiography), MRA (magnetic resonance angiography) or CBCT (cone-beam computed tomography) and 2D DSA (digital subtraction angiography) or 2D fluoroscopies. The X-ray acquisition can either be single plane or biplane. Rigid as well as non-rigid registration approaches for aligning the vessels from 3D/4D pre- or perioperative images with those from DSA or fluoroscopy have been described. These approaches update the 3D vessels position with regard to the C-arm but do not enable a continuous roadmap of the 3D vessels because continuous contrast agent injection during the intervention would be harmful to the patient. Calibration-based methods can be used when the 3D perioperative image and the 2D images are acquired with the same device. For example, if the 3D position of the C-arm is known accurately, it allows alignment of intra-operative 2D X-ray images with perioperative 3D images. Atasoy et al. [2] and Ruijters et al. [23] use C-arm information to update the registration between perioperative 3DRA (or CBCT) and 2D X-ray. This approach has been demonstrated to work accurately in cranial procedures with no head movement. Utilization in abdominal interventions, however, is hampered by the respiratory motion, which invalidates the initial alignment. Atasoy et al. [2] proposed a semiautomatic method to follow one moving region of interest selected by a physician during the intervention (a part of a catheter) and to update the registration with this information. The transformation model contains in-plane translation to correct for shifts caused by respiratory motion. In cardiac interventions, Ma et al. [12] used manual calibration-based methods to achieve an initial alignment and then used features such as diaphragm/heart border, tracheal bifurcation or the catheter to correct for breathing motion. Another method was proposed by Luan et al. [11] for oral cancer treatment. They track the catheter tip with an electromagnetic sensor, reconstruct the catheter path and then register it with a preoperative image. Although tracking the 3D catheter tip tracking is valuable, breathing motion may hamper the reconstruction of the path in, e.g., the abdomen. Unlike most of the other methods, our previous method [1] performs a 2D/3D catheter-based registration using a 3DRA and the complete catheter visible in the 2D X-ray images. It does not require 2D angiographic images nor user interaction for the initial alignment. However, computation times were not interactive, hampering interventional use.

The major contribution of our current work is to propose a method for generating an automatic continuous roadmap during abdominal catheterization using 2D/3D registration with single-plane 2D X-ray images and perioperative 3DRA. This paper is an extension of our previous work: The metric for alignment has been improved, the registration is faster, and the evaluation has been performed on a larger set of data, containing synthetic images, clinical images and additional evaluation metrics.

## Methods

The method is based on the registration of a 3D vessel tree with a 2D catheter shape. Therefore, in a preprocessing step, the arterial tree is extracted from the 3DRA image and the catheter shape position is determined from the single-plane fluoroscopic images. The extraction of the vessel tree itself is relatively straightforward for high-contrast 3DRA images. The segmentation of the catheter in the fluoroscopic images, albeit more challenging, has been subject of other studies [6, 16, 18, 19, 25, 28, 29]. These steps are not addressed in this paper.

**Fig. 2:**
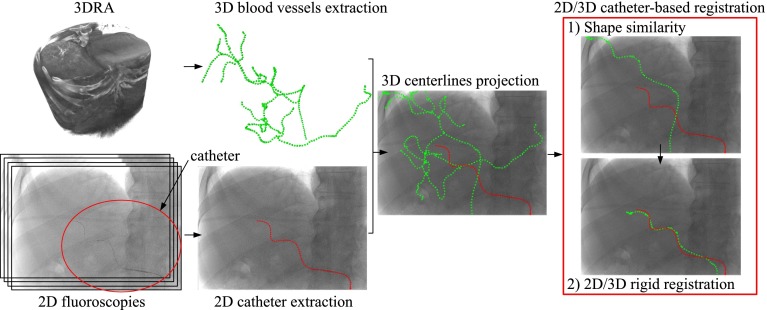
Global overview: vessels/catheter extraction and 2D/3D registration

Given the 3D vascular model and the 2D catheter centerline, the method consists of two steps (Fig. 2). First a shape similarity metric is used to find the vessel centerlines from the 3DRA that are most similar to the 2D catheter shape. Subsequently, a constrained 2D–3D registration is applied to find the corresponding rigid transformation between the 2D catheter and the 2D projections of the best-ranked vessel centerlines. In the following, we first define our coordinate systems and transformations, and then we describe each registration steps.

### Definitions

**Fig. 3:**
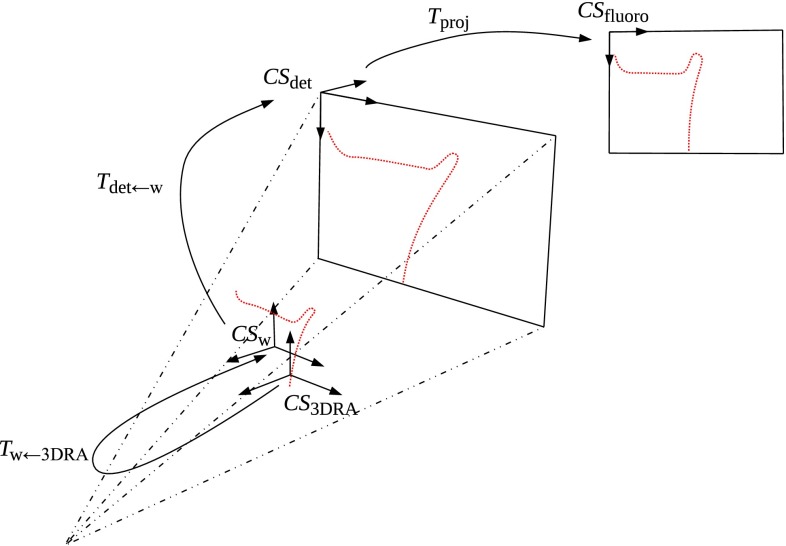
Coordinate systems and transformations of the C-arm space

We define the following coordinate systems (CS) for our setup in the intervention room (Fig. 3):

- $\hbox {CS}_{\mathrm {w}}$, denotes the world 3D CS, with the origin at the iso-center of the C-arm, and oriented along the C-arm in its default position
- $\hbox {CS}_{\mathrm {det}}$, the detector 3D CS (X-ray image plane)
- $\hbox {CS}_{\mathrm {fluoro}}$, the 2D CS of the fluoroscopic image
- $\hbox {CS}_{\mathrm {3DRA}}$, 3D CS of the 3DRA

Accordingly, the following coordinate transformations are defined:

- $T_{\mathrm {det\leftarrow w}}$, transformation from $\hbox {CS}_{\mathrm {w}}$ to $\hbox {CS}_{\mathrm {det}}$
- $T_{\mathrm {proj}}$, cone-beam projection from $\hbox {CS}_{\mathrm {det}}$ to $\hbox {CS}_{\mathrm {fluoro}}$
- $T_{\mathrm {w\leftarrow 3DRA}}$, transformation that aligns the 3DRA space with the patient, from $\hbox {CS}_{\mathrm {3DRA}}$ to $\hbox {CS}_{\mathrm {w}}$

$T_{\mathrm {det\leftarrow w}}$ and $T_{\mathrm {proj}}$ are known for each X-ray image because the geometry and orientation of the C-arm are known. $T_{\mathrm {w\leftarrow 3DRA}}$ is unknown and is the result of our registration.

With the projection function $F_\mathrm{proj}$ (in homogeneous coordinates):

1 $$\begin{aligned} F_\mathrm{proj}(p_{3D}, T) = T_{\mathrm {proj}} \cdot T_{\mathrm {det\leftarrow w}} \cdot T \cdot p_{3D}, \end{aligned}$$

we have a 3D point in the 3DRA space, $p_{\mathrm {CS_{3DRA}}}$, which can be projected on the fluoroscopic image space $\hbox {CS}_{\mathrm {fluoro}}$ using the following equation:

$$\begin{aligned} p_{\mathrm {CS_{fluoro}}} = F_\mathrm{proj}(p_{\mathrm {CS_{3DRA}}}, T_{\mathrm {w\leftarrow 3DRA}}). \end{aligned}$$

The catheter centerline extracted from a 2D fluoroscopic image is defined as an ordered set of $n_C$ points:

$$\begin{aligned} C_{\mathrm {2D}} = \{c_{\mathrm {1}}, c_{\mathrm {2}}, \ldots c_{\mathrm {i}}, \ldots , c_{\mathrm {n_C}}\}, \end{aligned}$$

where $c_{\mathrm {i}} \in \mathbb {R}^{\mathrm {2}}$ are 2D points at the center of the catheter in $\hbox {CS}_{\mathrm {fluoro}}$ and $c_{\mathrm {1}}$ denotes the tip of the catheter.

The blood vessel tree centerline extracted from the 3DRA is represented as a directed tree:

$$\begin{aligned} G_{\mathrm {3D}} = (\mathcal {P}, \mathcal {E}), \end{aligned}$$

where $\mathcal {P}$ is the set of 3D points on the centerlines of the vessels in $\hbox {CS}_{\mathrm {3DRA}}$ and $\mathcal {E}$ is the set of directed edges between points. The root of $G_{\mathrm {3D}}$ is in the aorta, and the branches of the tree are in the liver.

**Fig. 4:**
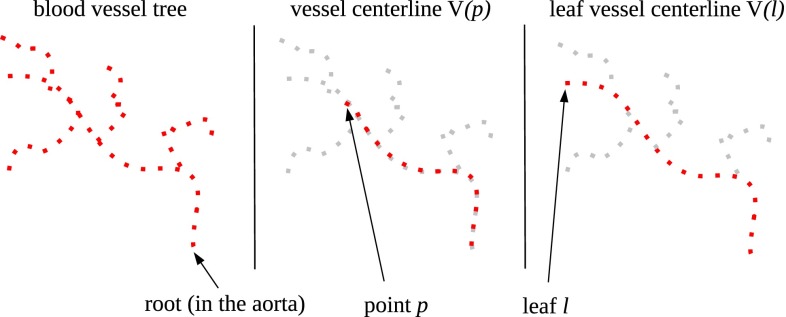
Terminology: blood vessel tree, vessel centerline and leaf vessel centerline

We define a vessel centerline $V(p)$ as an ordered set of points in $G_{\mathrm {3D}}$, from any point $p \in \mathcal {P}$ along the directed edges to the root (Fig. 4):

$$\begin{aligned} V(p) = \{p, p_1, p_2, \ldots p_i, \ldots , p_{n_P}\}, \end{aligned}$$

where $p_i \in \mathbb {R}^{\mathrm {3}}$ in $\hbox {CS}_{\mathrm {3DRA}}$ and $p_{n_P}$ is the root of $G_{\mathrm {3D}}$.

Similarly, we define the 2D projection of the 3D vessel centerline $V(p)$:

$$\begin{aligned} V_{proj,T}(p)= & {} \{F_\mathrm{proj}(p,T), F_\mathrm{proj}(p_1,T), \ldots \nonumber \\&F_\mathrm{proj}(p_i,T), \ldots , F_\mathrm{proj}(p_{n_P},T)\}. \end{aligned}$$

Additionally, we define $V_{proj,T}(p,u)$ with $u \in [0, V_l]$, a linearly interpolated version of the projected centerline, with $V_l$ the length of the projected vessel centerline $V(p)$.

### Shape-based vessel centerline selection

**Fig. 5:**
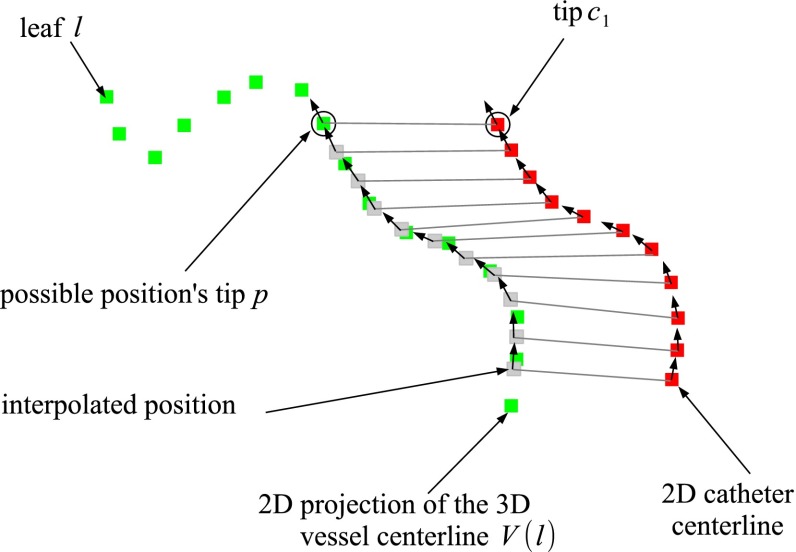
Discretized sum of dot products between tangents of catheter and the vessel centerline $V(p)$ with $p \in V(l)$

The registration is performed on a vessel centerline running from a leaf to the root (Fig. 4). Before performing the registration, the vessels that are the most likely to contain the catheter are selected. This selection is based on the shape similarity metric between the catheter and the projected 3D vessel. The metric quantifies the alignment of the tangent vectors of the catheter and the projected vessel. Therefore, it is not sensitive to the distance between centerlines. The underlying assumption is that the orientation of the vasculature changes little between the 3DRA and the fluoroscopy acquisition, which is valid for our application. The shape similarity for a vessel from a point $p \in \mathcal {P}$ is defined as:

2 $$\begin{aligned} S(p) = \int _0^{C_l} \! \overrightarrow{C}_{\mathrm {2D}}(u) \cdot \overrightarrow{V}_{\mathrm{proj},I_{4}}(p, u) \mathrm {d} u, \end{aligned}$$

where $C_l$ is the length of the 2D catheter, $I_{4}$ is the $4\times 4$ identity matrix, and $\overrightarrow{C}_{\mathrm {2D}}(u)$ (resp. $\overrightarrow{V}_{\mathrm{proj},I_{4}}(p, u)$) is the tangent of $C_{\mathrm {2D}}$ (resp. $V_{\mathrm{proj},I_{4}}(p)$) at the position $u$. $C_{\mathrm {2D}}(0)$ is the tip of the catheter, and $V_{\mathrm{proj},I_{4}}(p,0)$ is the possible location of the tip in the tree $G_{\mathrm {3D}}$. $S(p) \in [0, C_l]$ with $C_l$ denoting the maximum similarity. As the catheter centerline is represented as a set of points, the integral over S is approximated by summing the dot products over all catheter positions, thereby interpolating the corresponding vessel positions (Fig. 5).

In order to select the leaf vessel centerline for which the registration needs to be performed, for each leaf $l$, the maximum similarity over all points in V(l) is determined:

3 $$\begin{aligned} S_\mathrm{max}(l) = \max _{p \in V(l)} S(p). \end{aligned}$$

Based on the values of $S_\mathrm{max}$, we selected the $k$ leafs with largest $S_\mathrm{max}$ for the registration. When several leafs share the same common part with the catheter, only one is kept.

### Rigid 2D/3D registration with forward projection

To register the 2D catheter with the vessel centerline, we need to find the rigid transform $T_{\mathrm {w\leftarrow 3DRA}}$ that yields the best match with the 2D catheter in $\hbox {CS}_{\mathrm {fluoro}}$. We decompose the transformation as follows:

$$\begin{aligned}T_{\mathrm {w\leftarrow 3DRA}} = T_{\mathrm {w\leftarrow det}} \cdot T_{\mathrm {trans}} \cdot T_{\mathrm {det\leftarrow w}} \cdot T_{\mathrm {rot}},\end{aligned}$$

where $T_{\mathrm {rot}}$ is a rotation matrix with three unknowns (Euler angles, $\alpha$, $\beta$ and $\gamma$), and $T_{\mathrm {trans}}$ is a translation matrix with three unknowns ($x$, $y$, $z$) with the translations aligned in $\hbox {CS}_{\mathrm {det}}$. A translation along the projection axis in $\hbox {CS}_{\mathrm {det}}$ will only have a very minor effect in the projection. We therefore exclude $z$ from the registration parameters, leaving us with a five degrees of freedom transformation.

Our registration metric is the sum of distances between points on the catheter and closest points on the projected leaf vessel centerline. The catheter tip $c_1$ thereto is matched to the closest point of the projected vessel $V(l^\mathrm{sel})$, where $l^\mathrm{sel}$ is a leaf selected thanks to the shape similarity. The distance between the catheter tip and the vessel centerline $V(l^\mathrm{sel})$, given a rigid transformation T, is given by:

4 $$\begin{aligned} D_1(l^\mathrm{sel}, T) = \min _{p \in V(l^\mathrm{sel})} ||c_1 - F_\mathrm{proj}(p, T)||. \end{aligned}$$

Each next point of the catheter is similarly matched with a point of the projected vessel. To ensure continuity of the vessel (and simultaneously reducing computation time), the search range is limited to only a few points proximal to the point closest to the previous catheter point. Thus, let $p_\mathrm{prev} \in V(l^\mathrm{sel})$ be the point matched with $c_{i-1}$, then the distance to the subsequent catheter point $c_i$ is defined as:

5 $$\begin{aligned} D(c_i, l^\mathrm{sel}, T, p_\mathrm{prev}) = \min _{p \in [p_\mathrm{prev}, \ldots p_{\mathrm{prev} + h}]} ||c_i - F_\mathrm{proj}(p, T)||,\nonumber \\ \end{aligned}$$

where $[p_\mathrm{prev}, \ldots p_{\mathrm{prev} + h}]$ are the $h + 1$ consecutive points in $V(l^\mathrm{sel})$, starting at $p_\mathrm{prev}$, and $h$ is determined such that all points in that range are within a distance $d_\mathrm{max}$ of $p_\mathrm{prev}$.

**Fig. 6:**
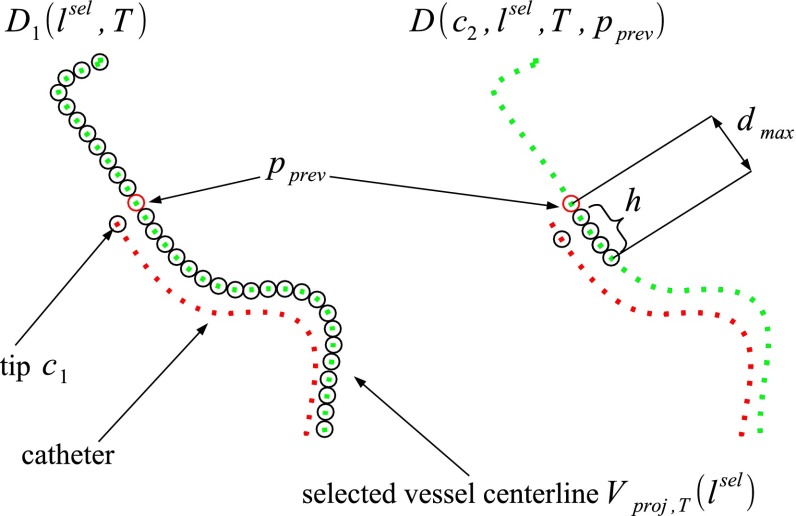
Registration metric $M(C_{2D}, l^\mathrm{sel}, T)$ with the first closest distances of the two points of the catheter centerline

**Fig. 7:**
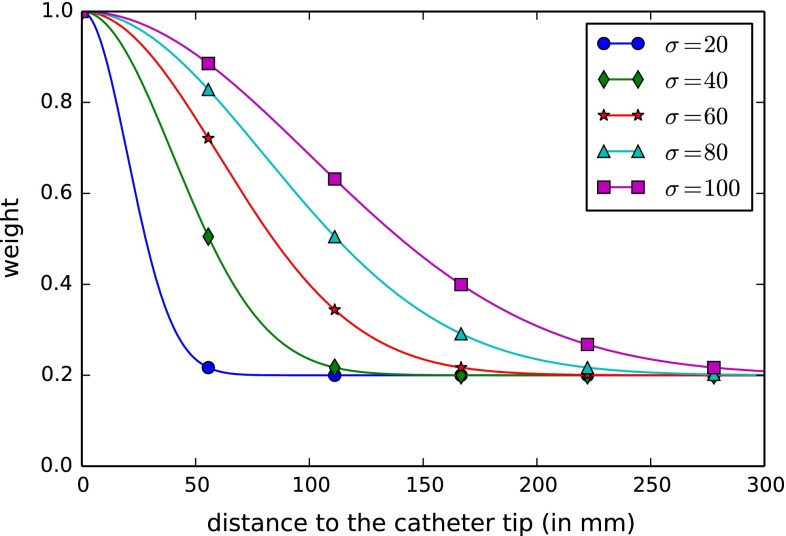
Weight function $W(x)$ to give more weight at the catheter tip with $\lambda = 0.2$ and various $\sigma$

Given these definitions, the final registration metric $M$ of our registration is a weighted sum of these distances (Fig. 6):

6 $$\begin{aligned}&M(C_{2D}, l^\mathrm{sel}, T) = D_1(l^\mathrm{sel}, T) \nonumber \\&\quad + \sum _{c_i \in [c_2, c_{n_{C}}]} W(||c_i, c_1||) \cdot D(c_i, l^\mathrm{sel}, T, p_\mathrm{prev}), \end{aligned}$$

where $W(x)$ is a weight function $\in [0,1]$ and $||c_i, c_1||$ is the length of the catheter between $c_1$ and $c_i$. As the registration accuracy close to the tip is more important than at the proximal part of the catheter, we use a weight to decrease the distance values that are further from the tip. We use a Gaussian with an offset:

7 $$\begin{aligned} W(x) = \lambda + (1 - \lambda ) \cdot e^{-\frac{x^2}{2\sigma ^2}}, \end{aligned}$$

where $\sigma$ is a parameter to control how fast the weight decrease (Fig. 7).

This metric $M$ has two advantages: first, it is fast because we only look for the closest point in a specific neighborhood; second, by only matching points that are locally connected the continuity of the vessel centerline is respected.

Lastly, the final transformation is the one with the smallest cumulative distance:

8 $$\begin{aligned} T_{\mathrm {w\leftarrow 3DRA}} = \underset{T}{{\text {arg}}\,{\text {min}}}\; M(C_{2D}, l^\mathrm{sel}, T), \end{aligned}$$

where $T$ represents the 5 degrees of freedom rigid transformation matrix.

Every selected leaf vessel centerline $V(l^\mathrm{sel})$ is registered, and the pair ($V(l^\mathrm{best})$, $T_{\mathrm {w\leftarrow 3DRA}}$) with the optimal similarity $M$ is kept.

## Experiments

We performed two series of experiments. In the first one, we used clinical data from TACE interventions. As we do not have a ground truth available in these data, we rather evaluate the registration on the alignment of the vessels distal to the catheter tip using angiography and we investigate the effect of varying parameter settings. In the second experiment, registration has been performed on the same clinical data, but with the catheter position simulated. The simulation allows us to have a ground truth for the catheter position. The error in the localization of the registered catheter tip position was used for evaluation.

### Data

**Fig. 8:**
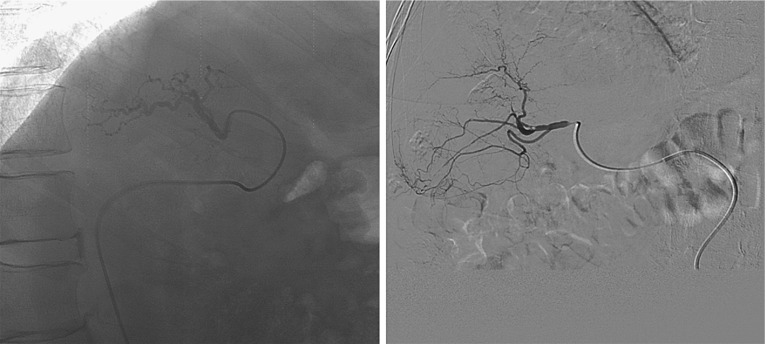
Fluoroscopy with contrast agent (*left*) and DSA (*right*)

We retrospectively acquired anonymized data of 19 TACE interventions (Table 1). The 16 first sets were acquired in the Erasmus MC, University Medical Center, Rotterdam, the Netherlands, between 2012 and 2014 in two different intervention rooms with angiographic C-arm systems (Xper Allura, Philips Healthcare, Best, the Netherlands). The last three sets were acquired in the Hôpitaux Universitaires Henri Mondor, Créteil, Paris, France, and the Ospedale di Circolo e Fondazione Macchi, Varese, Italy. For each intervention, we have a set of images consisting of one 3DRA image where the catheter was inside the hepatic artery, a set of fluoroscopic sequences with contrast agent and a set of digital subtraction angiographies (DSA) (Fig. 8). In these sequences, both the catheter and a part of the vasculature distal to the catheter tip are visible (by using the contrast agent). For each sequence, we selected the image with most of the vasculature visible and we manually annotated both the 2D catheter centerline and the 2D vasculature centerlines. The 3D arterial tree from 3DRA is segmented with a semiautomatic method based on thresholding and skeletonization [24].

**Table 1:** Number of clinical data

Patients	3DRA	Number of angios	Number of DSAs
01	Complete	4	2
02	Complete	2	3
03	Complete	0	2
04	Complete	1	4
05	Complete	3	1
06	Complete	2	2
07	Complete	5	1
08	Complete	2	3
09	Incomplete	4	3
10	Incomplete	2	3
11	Incomplete	3	2
12	Complete	2	2
13	Complete	1	4
14	Complete	0	2
15	Complete	3	2
16	Complete	3	5
17	Complete	1	2
18	Complete	1	0
19	Complete	0	2
Total	–	39	45

We divided our data in two different groups depending on the 3DRA acquisition: complete and incomplete acquisition. Incomplete acquisition occurs when the patient’s liver is not aligned with the C-arm rotation iso-center. In that case, the aorta and the hepatic artery are not visible in the 3DRA which hampers the registration.

### Implementation

The method described was implemented in C++ and run on a computer with a 3.4 Ghz Intel Core i7. We set $k$, the number of selected leaf vessel centerline to register, to 5.

In order to minimize our metric, we evaluated two different optimizers: a brute force and the Powell optimizer [20]. The brute force is exhaustive and is more likely to find the global minimum, whereas Powell is faster but because of its local search is more likely to converge to local minimum.

**Fig. 9:**
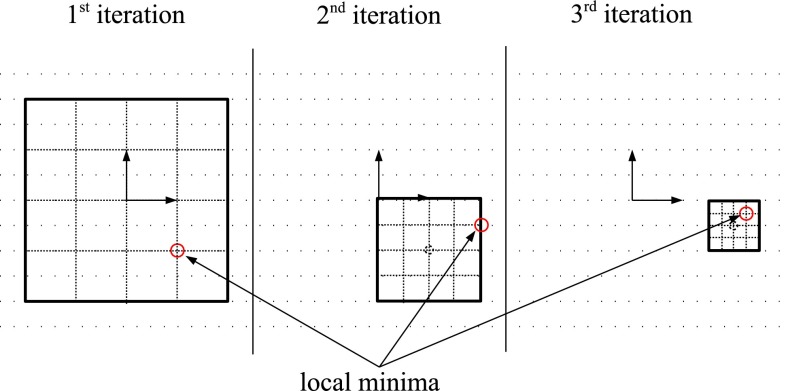
Brute force optimizer in a 2D space with $n = 3$ iterations, reduction coefficient $c = 0.5$, number of steps $d = 5$ and initial interval size $x, y = \pm 2$

Our brute force optimizer has $n=7$ iterations, and for each iteration $i$, the search space is centered at the minimum found in iteration $i - 1$ with an interval size $s_i = c*s_{i-1}$ whereby coefficient $c$ was set to 0.5 (Fig.  9). Each dimension in the search space interval is subdivided in $d=7$ steps. We set the initial intervals $s_0$ of our brute force search to $\pm$50 mm for $x$ and $y$ and $\pm$7$^\circ$ for $\alpha$, $\beta$ and $\gamma$. These intervals are sufficiently large to capture breathing motion.

For the Powell optimizer, we use a two-stage approach. We first optimize the in-plane translation and subsequently use that translation to initialize the full 5 degrees of freedom registration.

### Clinical data and parameter optimization

In the first experiment, we investigated optimal parameter settings for the method and evaluated how well the resulting registration aligns the vasculature distal to the catheter tip. To determine optimal parameter settings and evaluate the effect of changing parameters, we applied the method with a large set of different settings ($\lambda$, $\sigma$ and $d_\mathrm{max}$) in a leave-one-out cross-validation scheme (determine the optimal parameter values over the set containing all patients except the one on which the evaluation is done). We tested the following settings; $\lambda$: $0$, $0.1$, $0.2$, $0.3$; $\sigma$: $20$, $40$, $60$, $80$, $100$; $d_\mathrm{max}$: $10$, $20$, $30$, $40$, $50$ mm. Because the 2D catheter centerline and 3D blood vessel tree are discretized, we also investigated the effect of using different samplings: $1.5$, $3$ and $6$ mm between each point. Finally, computation time is recorded.

**Fig. 10:**
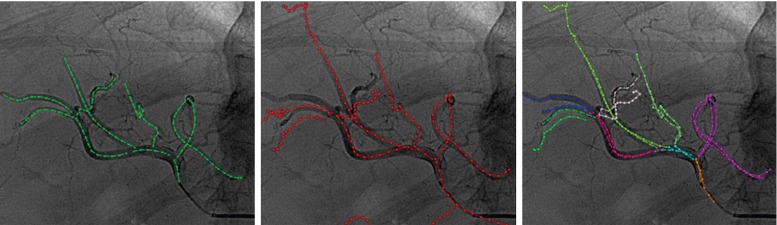
2D vasculature from contrast agent (*left*), 2D projection of 3DRA vasculature after manual registration (*center*) and manual-paired vessels (*right*): Same labels have same color vessels

**Fig. 11:**
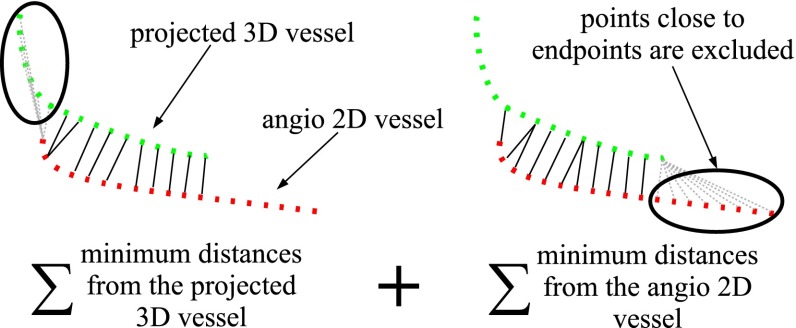
Closest corresponding points distance between paired vessels

As we do not have a ground truth for the registration, we used the vasculature visible on the angiographies as a reference. Thus, for validating, we compared how well the projected 3DRA matches the arteries visible in the projection images. To this end, we projected the 3DRA vasculature on the 2D image with the registered $T_{\mathrm {w\leftarrow 3DRA}}$ and we computed the closest corresponding points distance between the projected 3D vasculature and the 2D vasculature. The most relevant region for the roadmapping is the area close to the catheter tip; we therefore only evaluated in a circular region (3 cm radius) around the catheter tip. Before computing the distance, we manually labelled the vessels such that distances are computed between corresponding vessels (Fig. 10). To prevent bias in this assignment, the manual annotation was done without registration, thus only using the initial projection of the 3DRA. To aid in the annotation, the observer could manually register 3DRA and 2D vasculature by changing translations and rotations of the 3DRA. Vessels that cannot be manually adequately linked were not used in the evaluation. Using the labelled corresponding vessels, we computed the closest corresponding points distance for each pair of vessels (excluding distances to endpoints). The distance was computed both from the 2D angio-vessel and from its registered projected 3D vessel pair (Fig. 11). Our evaluation metric for one image is the average of distances over all pairs of vessels.

### Clinical data with a simulated catheter

When evaluating the registration on patient data, no accurate ground truth registration is available. We therefore also evaluated our method on simulated data with a known ground truth. To stay as close as possible to the reality, our simulation was based on the clinical data (Table 1) where we used the 3D extracted vessel tree in the 3DRA registered space (using $T_{\mathrm {w\leftarrow 3DRA}}$) as well as the fluoroscopic sequences with their projection information. We choose the position of the 3D simulated catheter tip in the 3D vessel tree such that it matches the catheter tip in the fluoroscopic image after a registration of the 3D vessel tree to the fluoroscopic image. Next, we extract a 3D simulated catheter centerline following the 3D vessel path from the tip to the root and project it on the fluoroscopic image using the angles from the fluoroscopic sequences acquired. Those ground truth projections are used to quantify the accuracy of the registration results. To simulate a smooth catheter that may be stretching the vessel and that may be partially outside the vasculature, we smooth the 2D projection centerline with a Gaussian kernel (with a standard deviation $\sigma _\mathrm{simu}$).

We applied random transformations $T_{\mathrm {w\leftarrow 3DRA}}$ to the 3DRA volume, divided over three sets depending on the magnitude of the transformations and the Gaussian smoothing parameter (Table 2).

**Table 2:** Randomizations for the simulation experiments

	Slight	Moderate	Large
Translation $x$ (in mm)	$[-30, 30]$	$[-30, 30]$	$[-30, 30]$
Translation $y$ (in mm)	$[-20, 20]$	$[-40, -20]\cup [20, 40]$	$[-50, -40]\cup [40, 50]$
Translation $z$ (in mm)	$[-30, 30]$	$[-30, 30]$	$[-30, 30]$
Rotation $\alpha , \beta , \gamma$ (in $^\circ$)	$[-6, 6]$	$[-6, 6]$	$[-6, 6]$
Catheter smoothing $\sigma _\mathrm{simu}$ (in mm)	$[1, 5]$	$[5, 10]$	$[10, 15]$

**Fig. 12:**
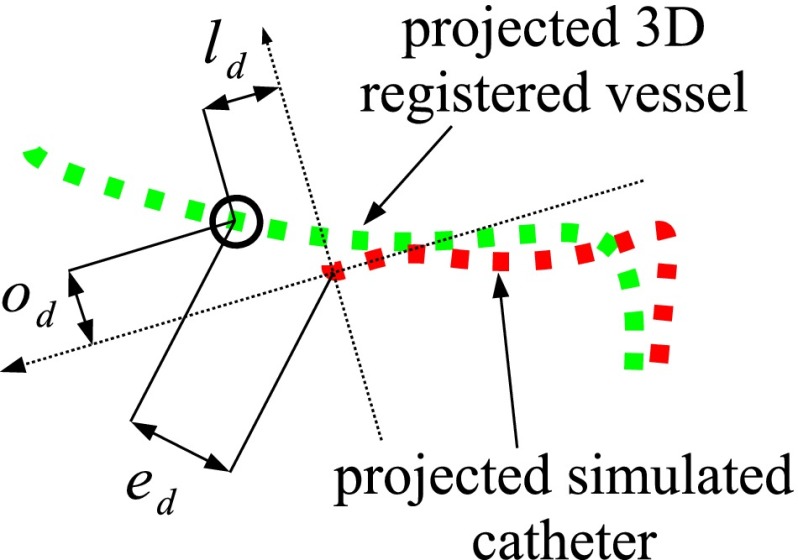
Evaluation of the registered tip position with $e_d$ the Euclidean distance, $l_d$ the longitudinal distance and $o_d$ the orthogonal distance

In this experiment, we used the algorithm parameters obtained in the previous leave-one-out cross-validation. We quantified the Euclidean distance $e_d$ between the known projected 3D tip and the registered projected 3D tip. We also computed the longitudinal $l_d$ and orthogonal $o_d$ distances from the point of view of the known tip (Fig. 12).

## Results

### Clinical data and parameter optimization

The results of the leave-one-out cross-validation with the two optimizers Powell and brute force, 3 mm sampling and all images are presented in Table 3. The optimal parameter settings are consistent over the leave-one-out experiments. Based on these results, unless noted otherwise, we used 3 mm sampling and with Powell: $\lambda = 0.2$, $\sigma = 20$, $d_\mathrm{max} = 40$ mm and with brute force: $\lambda = 0.1$, $\sigma = 80$, $d_\mathrm{max} = 20$ mm.

**Table 3:** Optimal settings for Powell (left) and brute force (right) with 3 mm sampling after a leave-one-out cross-validation

Patients	$\lambda$	$\sigma$	$d_\mathrm{max}$	Patients	$\lambda$	$\sigma$	$d_\mathrm{max}$
$01,02,03,04,05,06,08,09,10,11,12,13,14,15,16,17,19$	0.2	20	40	$02,03,04,05,06,07,08,09,11,12,13,14,15,16,17,18$	0.1	80	20
07	0.1	40	40	01	0.2	80	10
18	0	40	20	10	0.1	80	30
				19	0.1	40	20

**Fig. 13:**
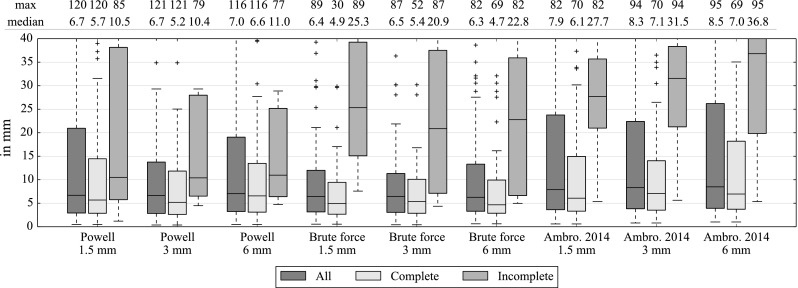
Average distance between paired vessels (in 3 cm radius from the tip) for each image after registration (in mm). Comparisons between Powell, brute force optimizer and our previous method Ambrosini et al. [1]

The average paired vessels distance results are summarized in Fig. 13. Results are grouped depending on the 3DRA acquisition. Compared with our previous method [1], using the same sampling, the new method has a median that is smaller. Also the brute force optimizer performs better than Powell and is more robust. With the complete 3DRA set, medians are around 5 mm for brute force and 6 mm for Powell. Varying the sampling density between 1.5 and 6 mm does not clearly affect the accuracy.

In Table 4, we present the distribution of the best registered leaf vessel centerline $V(l^{\mathrm {best}})$ among the $k$ ranked and selected leaf vessel centerlines. This table shows that the best registration result is generally obtained with the leaf vessel centerlines that ranked best using the shape-based metric $S_\mathrm{max}$. This demonstrates that the metric can effectively be used to reduce the number of potential vessels to register. The low percentage for the fifth ranked vessel also suggests that the choice of $k = 5$ is a good compromise between registration speed and the robustness of the method. The first ranked leaf vessel centerline is also the one giving the best registration for 39–49 % of the images.

**Table 4:** Distribution of the best registered leaf vessel centerline $V(l^{\mathrm {best}})$ among the $k=5$ ranked and ordered selected leaf vessel centerlines; with the optimal settings and the complete 3DRA set

	Sampling (in mm)	1st leaf (%)	2nd leaf (%)	3rd leaf (%)	4th leaf (%)	5th leaf (%)
Powell	1.5	40	29	10	8	13
Powell	3	49	24	12	9	6
Powell	6	45	24	10	16	5
Brute force	1.5	46	21	9	16	8
Brute force	3	39	24	8	19	10
Brute force	6	39	22	12	17	10

**Fig. 14:**
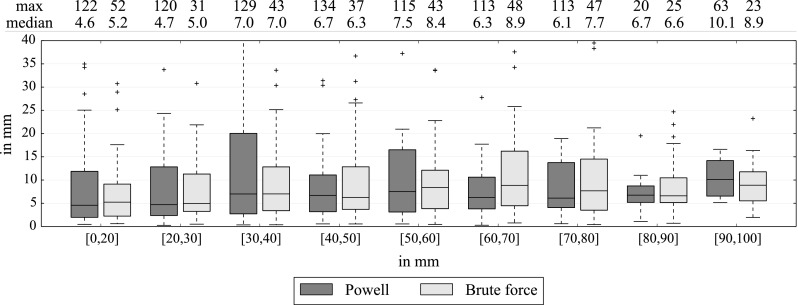
Average distance between paired vessels for all images (in mm) with optimal settings, 3 mm sampling and the complete 3DRA set. Paired vessels are grouped following their distance from the catheter tip (from 0 to 100 mm)

Figure 14 shows the paired vessels distance after the registration as function of the distance from the catheter tip. The median becomes less accurate after 3 cm between 1 and 5 mm.

**Fig. 15:**
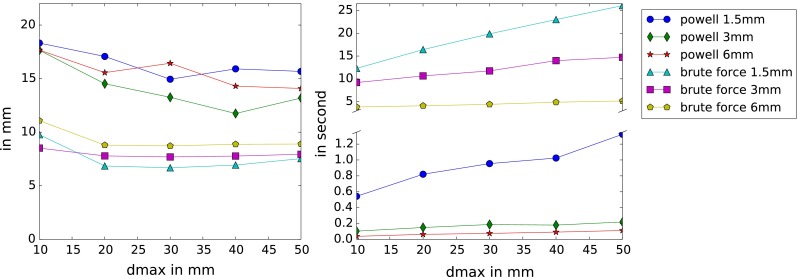
Average distance between paired vessels for all images in mm (*left*) and average time in second (*right*) according to the neighborhood distance $d_\mathrm{max}$, with optimal settings and the complete 3DRA set

The sampling and the local distance $d_\mathrm{max}$ in the metric $M$ are the parameters that affect the computation time. We show in Fig. 15 the relation between accuracy and computation time. Brute force is slower than Powell optimization. Our previous method [1] did the registrations with the samplings: 1.5, 3 and 6 mm in 95, 25 and 10 s, which is at least twice as slow as the brute force of our current approach. After 20 mm, $d_\mathrm{max}$ does not seem to change the accuracy with brute force. Powell is less stable with both the sampling and $d_\mathrm{max}$.

**Fig. 16:**
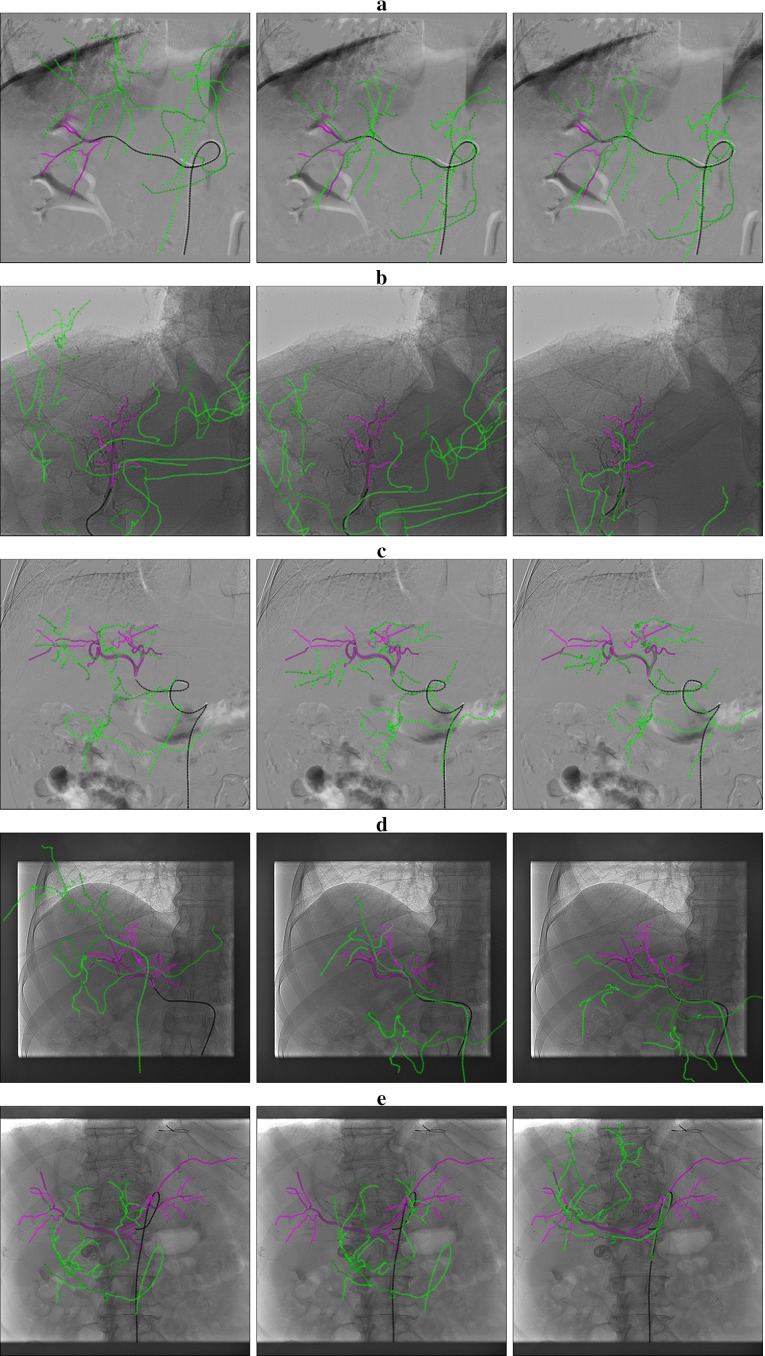
Projection of the 3DRA blood vessel (in *green*) with the catheter (in *black*) and the contrast agent (in *purple*). Initial position (*left*). Registered position with Powell (*middle*). Registered position with brute force (*right*). **a** The registration is correct. Here the catheter is long enough to give information. **b** The catheter part is too short. Powell registered with a good distance metric but the result is wrong. Brute force is correct. **c** The catheter tip position is correct for both optimizers. The vessels and catheter deformation prevent to have a perfect match. **d** Here the distance metric and the tip is correct with both optimizers but brute force rotates too much. **e** As a long part of the aorta is missing in the 3DRA, Powell stops in a local minimum while brute force is more exhaustive and reach the global minimum

Figure 16 shows examples of correct and incorrect registrations. We note that when there is a small part of the catheter visible on the image, the optimizers are more likely to yield misregistrations because of the lack of information. A correct tip position and distance metric $M$ do not imply a perfect match of the vasculature due to the deformation of the liver and the catheter.

We visually inspected all registrations from the complete 3DRA set with optimal settings and labelled them as correct, visually close and incorrect for both the registered tip and the registered vessels distal to the tip (Table 5). Visually close implies that the registration is sufficient to know where the catheter is in the 3D vasculature, while incorrect is of no use for the intervention. For each incorrect case, we also report the likely cause of failure (Table 6). The two main reasons of wrong registrations are a too small part of the catheter visible in the 2D X-ray image and large deformation of both the catheter and the vessels distal to it.

**Table 5:** Visual registration results with optimal settings, 3 mm sampling and the complete 3DRA set

	Powell (%)	Brute force (%)
*Selected registered vessel*		
Correct	84	94
Incorrect	16	6
*Match angio/registered vessels*		
Visually correct	38	35
Visually close	30	52
Incorrect	32	13
*Registered tip*		
Visually correct	59	73
Visually close	20	22
Incorrect	21	5

**Table 6:** Registration error details among incorrect match with optimal settings, 3 mm sampling and the complete 3DRA set

	Powell (%)	Brute force (%)
Small catheter part visible	40	38
Large vessels and catheter deformation	25	25
Catheter shape not sufficiently distinctive	5	12
Rotate too much to best fit the catheter		25
Powell stops in a local minimum	20	
Catheter only in the aorta (missing information)	5	
Large part of the aorta is not visible in the 3DRA	5	

### Clinical data with a simulated catheter

**Fig. 17:**
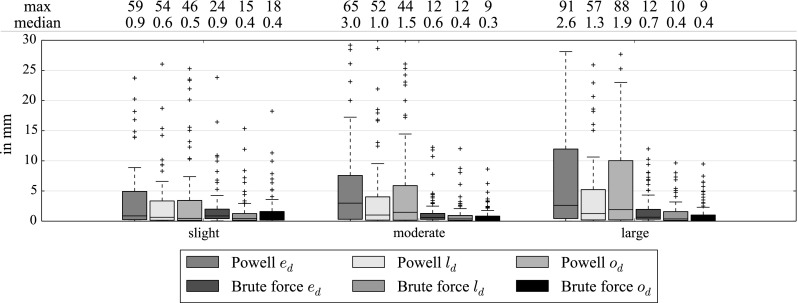
Euclidean distance $e_d$, longitudinal distance $l_d$ and orthogonal distance $o_d$ between the real tip and the registered one (in mm) with no catheter smoothing, 3 different simulations (Table 2), optimal settings and 3 mm sampling

**Fig. 18:**
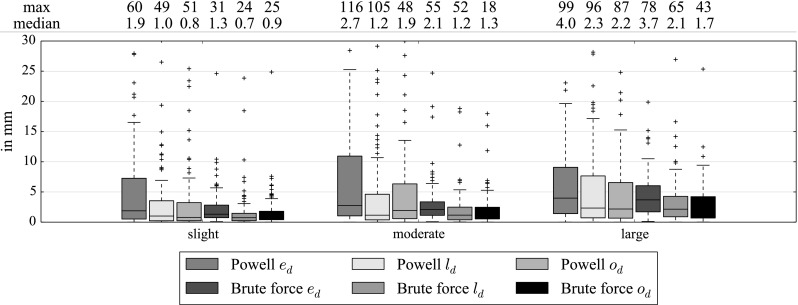
Euclidean distance $e_d$, longitudinal distance $l_d$ and orthogonal distance $o_d$ between the real tip and the registered one (in mm) with catheter smoothing, 3 different simulations (Table 2), optimal settings and 3 mm sampling

Figure 17 shows the distance between the real tip in the simulated catheter (without smoothing) and the tip after registration. Figure 18 shows the results with catheter smoothing. Without catheter smoothing, for the brute force optimizer, the median of the Euclidean distance $e_d$ is below 1 mm whereas for Powell the distance is below 3 mm. With catheter smoothing, the registered tip is less accurate and less robust with both Powell and brute force optimization. The longitudinal and orthogonal distance are similar with slight, moderate or large transformation. Overall, the longitudinal distance is slightly more robust than the orthogonal.

## Discussion and conclusion

We proposed and evaluated a method that enables a continuous roadmap during abdominal catheterization. The method registers a 3D vessel model obtained from 3DRA imaging using a catheter that is extracted from single-plane 2D X-ray images with or without contrast agent. The method first selects the vessels using shape similarity and then rigidly registers the selected vessels to the catheter.

With the complete 3DRA set and optimal settings, the median of average paired vessels distances of the roadmap distal to the catheter tip and within a radius of 3 cm from the tip is 5.4 mm for the brute force optimizer and 5.2 mm for the Powell optimizer. The first selected vessel during shape similarity is used more than 39 % in the final registration and the second more than 21 %.

We investigated two optimizers for the registration approach: brute force and Powell. In our setup, with $<$200 ms computation time on average, the registration is real time with the Powell optimizer and a 3- or 6-mm sample interval. Though the brute force optimizer is slower, it could be improved with parallelization and a dedicated implementation. The brute force optimizer tends to be more accurate and robust than the Powell optimizer. Powell is more sensitive to the initial position of the registration (ends up in local minima) as well as the length and distinct shape of the catheter.

The simulation experiments with catheter deformation demonstrate that the registration is robust for both optimizers with slight deformation. They also show that larger deformation leads to less accurate registration. In the simulated data, the longitudinal distance from the tip shows how well the tip is registered along the catheter direction. This distance is more significative than the orthogonal and is slightly more robust.

An important source of error in our experiments was the lack of vessel information in the 3DRA (especially missing the aorta and the hepatic artery), partly caused by the retrospective nature of our study. Optimization of the 3DRA acquisition protocol could remedy this. Another source of registration errors is the lack of information in the 2D X-ray because only very short part of the catheter is visible. This shows the limitation of the method working with no prior knowledge other than the current image. This may be addressed, during the intervention, by slightly increasing the field of view, moving the patient table, or adding more a priori knowledge into the registration such as previous image registration transformations. If we take into account the previous registered transformations and the table motion (which in principle could be obtained from the C-arm system, but is not available in our acquired fluoroscopic images), the registration should have a better initialization and thus use a smaller search space and both Powell and brute force optimizers will perform more robustly while reducing computation time.

Most related 2D/3D registration methods register angiography with CTA or 3DRA. As the complete vasculature is visible on both 2D and 3D images and non-rigid registration is performed, they reach submillimeter accuracies. Our method, dealing only with the catheter visible on the 2D image, has lower accuracy. However, we are interested in improving guidance, and the fusion provides a continuous roadmap of sufficient accuracy to the clinician to reliably estimate the catheter tip position in the 3D vasculature. As far as we know, in abdominal studies, the presented method can be compared only with the method proposed by Atasoy et al. [2]. They evaluate their method with the overlap of the 3DRA vessels onto the catheter. In our case, the overlap is our distance metric so a comparison will be biased toward our method.

During abdominal catheterization, knowledge of the position of the tip in the 3D vasculature is of crucial importance. Table 5 shows a small percentage of incorrect registered tip positions. This implies that if we use the presented fusion method as a roadmap, combining any of the optimizers, the resulting fused visualization is sufficient to guide the interventionists in localizing the tip and identifying the subsequent bifurcations, also in case of slight misalignment.

A robust automatic 2D catheter segmentation is required after initialization to integrate our method into the interventional workflow. The accuracy of the segmentation will influence the registration method. For example, Heibel et al. [6] obtain a median error of real-time automatic catheter tracking $<$1.5 pixels for abdominal fluoroscopies. Those results are sufficiently accurate for our registration.

Registration studies often lack ground truth for clinical data. In our case, this also prevented us to evaluate the accuracy of the registration method directly. However, both the simulation experiments and the validation with angiographic images demonstrate the good performance of the method.

In our current setup, each registration is independent from previous registrations. During continuous roadmapping, only slight motion should occur between two registrations. In the future, we intend to use previous registration results to further improve the robustness (especially when the visible catheter part is too small to do an accurate independent registration) and to limit the computation time by reducing the space search. A source of registration errors was due to large vessels and catheter deformation. A non-rigid registration to match the catheter deformation could also improve the accuracy close to the catheter tip.

To conclude, we presented a catheter-based registration method to fuse continuously 3DRA roadmap arterial tree onto 2D fluoroscopic images. We evaluated our work with clinical and simulated data demonstrating an efficient shape similarity and a median accuracy, evaluated on close by vessels, of 4.7–6.6 mm and below 4 mm on simulated experiments.
